# Algorithm for asthma diagnosis and management at Chitungwiza Central Hospital, Zimbabwe

**DOI:** 10.11604/pamj.2020.37.85.19543

**Published:** 2020-09-24

**Authors:** Pisirai Ndarukwa, Moses John Chimbari, Elopy Nimele Sibanda

**Affiliations:** 1 University of KwaZulu Natal, College of Health Sciences, School of Nursing and Public Health, Durban, South Africa,; 2 Asthma, Allergy and Immunedsyfunction Clinic, 113 Kwame Nkrumah Ave, Harare, Zimbabwe

**Keywords:** Algorithm, asthma, asthma diagnosis, asthma management, Zimbabwe

## Abstract

**Introduction:**

asthma is a chronic inflammatory and a heterogeneous condition of respiratory system whose pathogenesis is linked with variable structural changes. The clinical manifestation of asthma includes attacks of breathlessness, cough, chest tightness and wheezing. Provision of basic equipment and test for asthma diagnosis and access to essential medicines by asthmatic patients reduces morbidity and mortality rates. Significant progress has been made in the diagnosis and management of asthma in other countries but not in the health care delivery system in Zimbabwe. Therefore, the aim of this study was to develop algorithm for asthma diagnosis and management for Zimbabwe.

**Methods:**

a two stage Delphi model was used to collect data in order to develop an algorithm of asthma diagnosis and management. A baseline interview with 44 doctors was done to understand their experiences and knowledge regarding asthma diagnosis and management. We collected data using the KoBo Collect Toolbox installed on Android mobile phone and transferred the data to an Excel 2016 spreadsheet for cleaning. The data was qualitatively analysed and themes were constructed. These themes were further explored in stage two at an algorithm development workshop which was led by 4 medical expert panellists in order to develop consensus on the information to be included in the algorithms for asthma diagnosis and management. A total of 15 doctors and 30 nurses participated at the workshop.

**Results:**

doctors who attended the workshop described the challenges in asthma diagnosis and management that they experienced. These challenges were attributed to lack of basic equipment such as spirometers and Peak Expiratory Flow Meters and tests which included IgE tests, Skin Allergen Tests and RAST. Asthma diagnosis clinical history and management was based on the doctors' knowledge. The doctors indicated the need for refresher courses to update their knowledge on asthma diagnosis and enhance their diagnostic skills. A draft algorithm framework for asthma diagnosis was developed at the workshop and later refined by the core-research team. The final algorithm described in this paper was circulated for further contributions and endorsement by the asthma experts.

**Conclusion:**

we established the need for doctors to receive constant refresher courses on asthma diagnosis for upskilling. We recommend adoption by the Zimbabwe's Ministry of Health and Child Care of the asthma diagnostic algorithm we developed.

## Introduction

Asthma is a chronic inflammatory and a heterogeneous condition of respiratory system whose pathogenesis is linked with variable structural changes [1]. The clinical manifestation of asthma includes attacks of breathlessness, cough, chest tightness and wheezing. It is a major public health problem worldwide affecting 334 million people and a cause of 300 thousand death per year [1]. The prevalence of asthma in Australia is estimated to be above 25% and in the UK, it is between 20 and 25% [2]. In Zimbabwe the prevalence in the age group 18-45years is estimated to range from 0-10% [2]. The World Health Organisation (WHO) has reported that asthma is among the top 20 causes of death in Zimbabwe accounting for 0.89% of the total deaths [3]. A study by Salim, et al. reported asthma as a common condition in Zimbabwe which has been underreported [4].

Significant progress has been made in the diagnosis and management of asthma elsewhere [5,6]. These studies have developed guidelines for diagnosis and management of asthma that are more applicable to developed countries. Sustaining quality of care for asthma is difficult in low- and middle-income countries (LMICs) where there are no guidelines for asthma diagnosis and management [7]. A review of studies in Nigeria on asthma management pointed to multifaceted problems that included lack of standard diagnostic equipment, unavailability of local guidelines for diagnosis and management of asthma and lack of knowledge of health care providers on management asthma [8]. Although asthma is being diagnosed and managed in Zimbabwe, the country does not have an effective strategy for diagnosis and management of it [9]. All the Zimbabwean hospitals use the symptom based approach to diagnose and manage asthma and this is usually performed by experienced clinicians [10]. The symptom based approach is problematic because asthma overlaps with other respiratory conditions. We therefore developed a specific algorithm for asthma diagnosis and management that we believe will contribute to the improvement of the quality of life of asthma patients.

## Methods

**Study design and population:** this study was conducted using a two stage Delphi model [11]. A Delphi model is a more robust method that utilises expert judgements, and compares these judgements in several rounds with the aggregate judgements of other participating experts, until consensus on pre-specified criteria is reached [12,13]. Data was collected from the 27^th^of November to the 20th December 2018 at Chitungwiza Central Hospital situated about 30 kilometres south east of Harare. The hospital serves a population of over a million residents of Chitungwiza City and surrounding areas [14]. The 500 bed capacity hospital has casualty and outpatients departments where asthma cases are diagnosed and managed. The study participants were hospital based doctors and nurses.

**Sample size:** stage 1: forty-four clinicians (consisting of 41 General Medical Officers and the 3 Specialist Physicians) working in at Chitungwiza Hospital were purposively sampled to provide baseline knowledge for asthma diagnosis and management. Stage 2: Forty-five participants who included 15 doctors and 30 nurses participated at an algorithm development workshop moderated by four medical experts

**Data collection and analysis:** data was collected using the Kobo Collect Toolbox. KoBo is an open source platform that is used to collect and analyze data [15]. The data collector was trained on how to conduct interviews and recording responses in KoBo Collect Toolbox. Data collection was in two stages; stage 1 was an interview with doctors and stage 2 was an algorithm development workshop.

**Stage 1: data collection using the modified Delphi model (interviews):** in stage 1, forty four doctors were interviewed by an experienced doctor with relevant knowledge on asthma diagnosis and management using a semi-structured questionnaire. The questionnaire was divided into two sections; (i) experiences and knowledge of clinicians regarding asthma diagnosis and (ii) doctors´ knowledge regarding asthma management. Data analysis grouped the information collected into qualitative themes that were used at the algorithm development workshop. This formed the baseline results of our study from which the preliminary results we build on.

### Stage 2: algorithm development workshop

**Stage 2(a): brainstorming:** the brainstorming session discussed the challenges experienced in the diagnosis and management of asthma following a presentation on the possible pathways of asthma diagnosis and treatment made by an asthma specialist and another presentation on preliminary findings of interviews held with doctors and asthma patients.

**Stage 2(b): group work:** participants at the workshop were divided into two groups; group 1 crafted a draft algorithm framework for asthma diagnosis and group 2 crafted a draft algorithm for asthma management. The two groups presented their algorithms in a plenary session.

**Stage 2(c): plenary session:** during the plenary session, each group presented their draft framework and discussions ensued with facilitation and moderation by subject experts. Following plenary contributions consensus was reached on the draft algorithms.

**Ethical considerations:** permission to conduct the study was granted by the Biomedical Research Ethics Committee of the University of KwaZulu-Natal (BE613/18) as well as the Medical Research Council of Zimbabwe (A/2352). Gatekeeper permission to do the study was given by the Ministry of Health and Child Care, Zimbabwe; and the Chitungwiza Central Hospital before commencement of data collection. All the doctors who participated in this study signed a consent form after reading and understanding the conditions of the study. The mobile electronic devices that we used for data capturing did not capture personal identification details; each doctor who participated in this study was assigned to a code. Those nurses and doctors who agreed to participate in the algorithm development workshop did so voluntarily and were free to terminate their participation at any time during the process of the workshop.

## Results

There were two distinct results in this study; (i) Preliminary results on doctors´ knowledge on asthma diagnosis and management (interviews) and (ii) algorithm on asthma diagnosis and management.

### Preliminary results on doctors´ knowledge on asthma diagnosis and management (interviews)

**Experiences and knowledge of challenges on asthma diagnosis:** the experience of asthma diagnosis and management by most of the doctors interviewed ranged between 2 and 6 years. However, the 3 specialist physicians had experiences which were 7 years, 13 years and 21 years as specialist physicians. All clinicians (Government medical officers (GMO) and specialist physicians) highlighted that asthma diagnosis and management was inadequately resourced. They said they experienced some difficulties in diagnosing asthma due to overlapping symptoms which mimicked other respiratory conditions prevalent in the area. The clinicians also felt that their competencies in diagnosing asthma were limited. One specialist physician said: “*Most of the challenges that I face in coming up with asthma diagnosis include non-availability of equipment such as spirometry, Peak Flow Meters and most embarrassing is that I am not even able to perform a Bronchial provocation test*.” Another clinician (GMO) weighed in and said: “*Asthma has overlapping symptoms which makes it difficult to make an appropriate diagnosis. Also as clinicians we lack competencies in diagnosis because asthma follows mimicry of symptoms with other respiratory conditions such as COPD and emphysema*.”

**Knowledge on resolving challenges of asthma diagnosis:** all clinicians highlighted the need for basic equipment such as Asthma Control tests, Peak Expiratory Flow Meters and Spirometers. They highlighted the need to avail allergen tests like the IgE Serum Allergen tests. Most (93.2%) of the clinicians said that there was need for continuous medical education on asthma and availing algorithms for asthma diagnosis. A clinician (GMO) emphasized: “*Making asthma diagnosis protocol available in the clinician´s consultation room would be phenomenal in assisting the clinician to remember the key features to asthma diagnosis*.”

**Knowledge of cardinal signs of asthma:** all (100%) doctors showed knowledge of the cardinal signs of asthma diagnosis which they highlighted as sleep apnoea, wheezing, cough, chest tightness and shortness of breath. All (100%) doctors indicated that the asthmatic wheeze was polyphonic in nature. All (100%) doctors classified the shortness of breath according to World Health Organisation as mild, moderate and severe asthma. Only a few (27.3%) doctors identified a cough occurring between 02 00 and 04 00hours as indicative of asthma. Majority (72.7%) doctors gave varying responses of times when asthma patients coughed.

**Knowledge of risk factors for asthma:** all (100%) doctors knew of the risk factors for asthma and they indicated allergens like dust, perfumes, pollens and flowers as risk factors for asthma. All (100%) doctors highlighted respiratory infections, emotions, cold temperatures, exposure to atopy and exercises as risk factors for asthma. The doctors then grouped the risk factors as both indoor, outdoor, food and emotions (Table 1).

**Table 1:** risk factors for asthma

Risk factor	Frequency n (%)
Indoor and outdoor	3(6.8)
Indoor, outdoor and food	9(20.5)
Indoor, outdoor, food and emotions	19(43.2)
Indoor, outdoor and emotions	7(15.9)
Outdoor and food	2(4.6)
Outdoor, food and emotions	2(4.6)
Outdoor and emotions	2(4.6)

**Knowledge of asthma management:** all (100%) doctors acknowledged that their ability to manage asthma was basic and was constrained by non-availability of asthma medications, inability to use inhaler technology and lack of specific local guidelines for asthma management according to its severity. Seventy point five percent (70.5%) indicated the need for some refresher courses on asthma management. One clinician (GMO) said: “*I know that I am only managing asthma based on symptoms approach and this is not good for patients. We lack appropriate guidance on how to demonstrate use of inhaler medications which is resulting in our patients not recovering*.”

**Figure 1:**
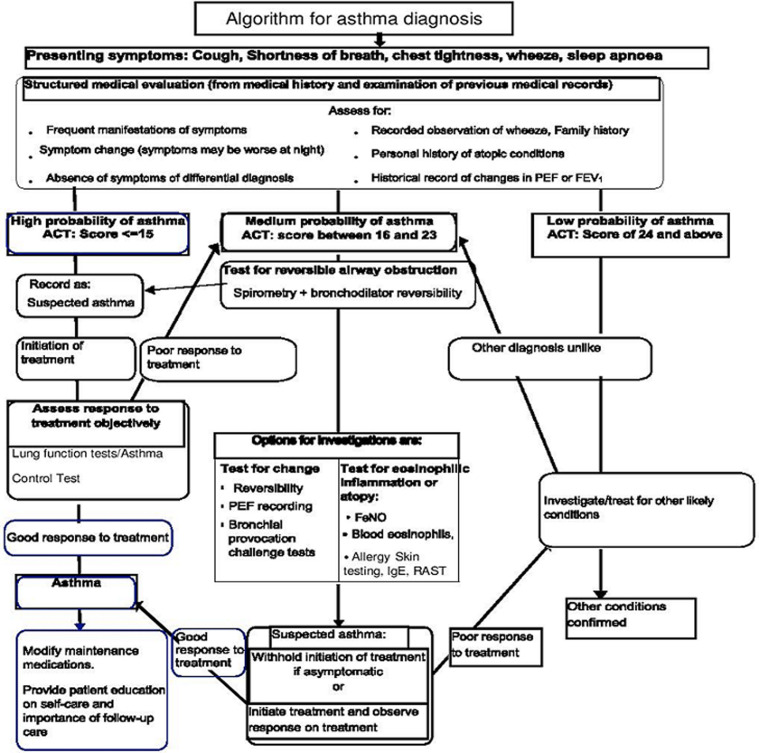
algorithm for asthma diagnosis

**Figure 2:**
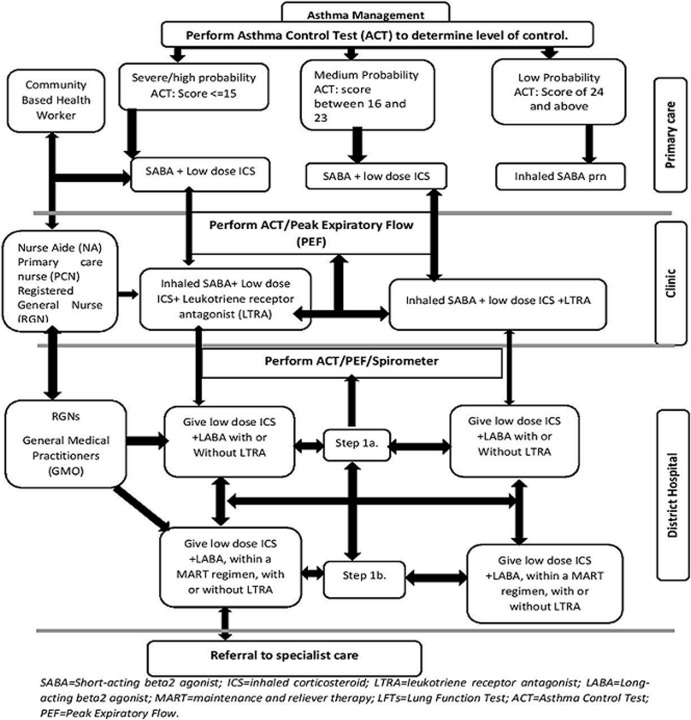
algorithm for asthma management

**Algorithms on asthma diagnosis and management:** the results of the algorithms are based on the algorithm development workshop facilitated by medical experts. Figure 1, Figure 2 below showed the algorithms for asthma diagnosis and asthma management respectively.

## Discussion

The results of our study indicated a serious lack of essential resources for asthma diagnosis including basic equipment such as Peak Flow Meters and Spirometers and allergen tests. The lack of these basic equipment and allergen tests can result in clinicians underdiagnosing asthma. Our findings concur with a systematic review of studies done by Onyedum *et al*. [8] who showed that the overall effect of diagnostic challenges will lead to under diagnoses, over diagnoses, misdiagnoses and sometimes undiagnosed/ unreported cases of asthma leading to increased morbidity and mortality due to asthma. Our study revealed that clinicians lacked the essential tools to differentiate between asthma and other respiratory conditions whose symptoms mimick asthma. Tanabe *et al*. [16] showed that asthma mimicked congestive heart failure which could present with wheezing and airway obstruction from pulmonary oedema and pulmonary vascular congestion. This indicates the need for health care providers to be supplied of basic diagnostic tools to be able to isolate asthma from other respiratory conditions. This has been indicated in the proposed algorithm that has been developed in the study. Similar sentiments on the need to consider various differential diagnoses in patients presenting with asthma-like symptoms have also been reported in a review by So *et al*. [17]. However, our study did not look at differential diagnosis.

Our study highlighted lack of continuous medical education as a factor that contributes to lack of knowledge by clinicians in asthma diagnosis and management. This is not unusual in Southern Africa as a study in Zambia also showed that a few health workers had received specialty training in asthma management [18]. Another study by Wood *et al*. [19] in Malawi showed that the health care providers lacked training and confidence in their own skill levels in management of NCDs including asthma. A study by Mardsen *et al*. [20] who indicated that there is need for strategies that increase education and awareness about asthma in order to improve disease management. The findings from these studies have clearly shown the need for continuous medical education.

Results from our study revealed lack of local guidelines for asthma diagnosis and management. In light of these findings, the study further developed an algorithm for diagnosis and management of asthma within our local context. The presence of local guidelines can have a positive impact in asthma diagnosis and management. The local guidelines can improve health care workers´ knowledge of diagnosing and managing asthma, thus reducing the risk of asthma morbidity and mortality. The algorithm for diagnosis we developed is the first of its kind for Zimbabwe and we hope that it will improve asthma case identification thus reducing morbidity and mortality rates associated with misdiagnoses and/or under diagnoses. The algorithm on management of asthma will reduce the likelihood of under treatment and/or mistreatment which will contribute to improved quality of life for asthma patients. Our study was limited to tertiary hospitals which may affect generalization of the results to other settings. The study was limited to health care providers and did not establish the opinion of the patients as they are the once who may potentially be adversely affected by misdiagnosis or mismanagement.

## Conclusion

This study established the need for health care providers to receive constant refresher courses on asthma diagnosis for upskilling. We recommend adoption by the Zimbabwe´s Ministry of Health and Child Care of the asthma diagnostic and management algorithms we developed.

### What is known about this topic

- It has been established that asthma diagnosis is poorly done by health care workers in the health care settings in Zimbabwe and parts of Africa.
- Prior studies in Low to Middle income countries (LMIC) have documented that health care workers lack knowledge on asthma diagnosis and management, but no study has been done to develop a tool that could be used to ease diagnosis challenges of asthma patients and management.

### What this study adds

- This study found that health care workers lacked knowledge of asthma diagnosis and management at Chitungwiza Central Hospital in Zimbabwe, therefore, continuous medical education was recommended as a strategy to improve health care workers' knowledge on asthma diagnosis and management.
- The study lead to the development of algorithms for asthma diagnosis and management that is hoped to reduce morbidity and mortality rates for asthma in Zimbabwe.

## References

[1] Hay SJLRM (2017). Global, regional, and national deaths prevalence disability-adjusted life years and years lived with disability for chronic obstructive pulmonary disease and asthma 1990-2015 a systematic analysis for the Global Burden of Disease Study 2015. Lancet Respir Med.

[2] Global Asthma Network (2018). The Global Asthma Report.

[3] World Health Organization (2017). CIA and individual country databases for global health and causes of death.

[4] Salim A, Bwakura T, Dzvanga N, Gordon S (2008). The epidemiology of respiratory disease in Zimbabwe.

[5] James DR, Lyttle MD (2016). British guideline on the management of asthma: SIGN Clinical Guideline 141 2014.

[6] Lougheed MD, Lemiere C, Ducharme FM, Licskai C, Dell SD, Rowe BH (2012). Canadian Thoracic Society 201.2 guideline update: diagnosis and management of asthma in preschoolers children and adults. Can Respir J.

[7] Global Asthma Network, New Zealand (2014). The global asthma report.

[8] Onyedum CC, Ukwaja KN, Desalu OO, Ezeudo C (2013). Challenges in the management of bronchial asthma among adults in Nigeria: A systematic review. Ann Med Health Sci Res.

[9] Asher I, Haahtela T, Selroos O, Ellwood P, Ellwood E, Global Asthma Network Study Group (2017). Global Asthma Network survey suggests more national asthma strategies could reduce burden of asthma. Allergol Immunopathol (Madr).

[10] National Institute for Health and Care Excellence (UK) (2017). Asthma: diagnosis and monitoring of asthma in adults, children and young people.

[11] Landeta J, Barrutia J, Lertxundi A (2011). Hybrid Delphi: A methodology to facilitate contribution from experts in professional contexts. Technological Forecasting and Social Change.

[12] Jones J, Hunter D (1995). Consensus methods for medical and health services research. BMJ.

[13] Minkman M, Ahaus K, Fabbricotti I, Nabitz U, Huijsman R (2009). A quality management model for integrated care: results of a Delphi and Concept Mapping study. Int J Qual Health Care.

[14] (2012). Zimbabwe Population Census: Zimbabwe National Statistics Agency.

[15] Palla F (2016). New technologies: mobile data collection system implication for wildlife management in Central Africa.

[16] Tanabe T, Rozycki HJ, Kanoh S, Rubin BK (2012). Cardiac asthma: new insights into an old disease. Expert Rev Respir Med.

[17] So JY, Mamary AJ, Shenoy K (2018). Asthma: Diagnosis and Treatment.

[18] Somwe Wa S, Jumbe-Marsden E, Mateyo K, Senkwe MN, Sotomayor-Ruiz M, Musuku J (2015). Improving paediatric asthma care in Zambia. Bull World Health Organ.

[19] Wood R, Viljoen V, Van Der Merwe L, Mash R (2015). Quality of care for patients with non-communicable diseases in the Dedza District, Malawi. Afr J Prim Health Care Fam Med.

[20] Marsden EJ, Somwe Wa S, Chabala C, Soriano JB, Vallès CP, Anchochea J (2016). Knowledge and perceptions of asthma in Zambia: a cross-sectional survey. BMC Pulm Med.

